# The Fifteenth International Congress of Medicine, Lisbon, 1906

**Published:** 1905-11-11

**Authors:** 


### THE FIFTEENTH INTERNATIONAL CONGRESS OF MEDICINE, LISBON, 1906.

The fifteenth meeting of the International Congress of Medicine will be held at Lisbon from the 19th to 26th April, 1906. The Executive Committee, guided by its energetic secretary, Professor Miguel Bombarda, is taking every precaution to overcome the difficulties which reduced the Madrid meeting to a state of chaos. There is every reason, therefore, to hope that the meeting will be at least as pleasant and well managed as the Congress at Paris, or as the recent meeting of the International Society of Surgery at Brussels. One of the chief difficulties in the matter of the Lisbon Congress is the accommodation of visitors, because the really first-rate hotels in the city are very few. The National Committee for Great Britain and Ireland have, therefore, made a wise departure. They have approached Messrs. Thomas Cook and Son with a view to charter a vessel which should convey the British members to Lisbon, serve them as an hotel during the Congress, and bring them back again to London as soon as it was over. Messrs. Cook and Son now announce that they have obtained the s.s. *Ophir* for this purpose. The boat is well known for its large size and first-rate passenger accommodation, and it was the vessel in which T.R.H. the Prince and Princess of Wales recently made a voyage to Australia. It is proposed that the *Ophir* shall leave London in the afternoon of Thursday, April 12—the day before Good Friday—and return to London early in the morning of Monday, April 30. The fares are fixed at 15-35 guineas per person, according to the position of the cabin, including food and free communication at frequent intervals between the ship and the shore. Whilst the vessel lies in the Tagus, the harbour authorities have allotted to it the berth lately given to the Royal yacht when H.M. the Queen, with H.R.H. the Princess Victoria, paid a visit to Lisbon. This berth is about 500 yards from the shore, and is in deep water with a rapid stream. It is hoped that the employment of the s.s. *Ophir* will obviate the necessity of making arrangements for lodging in Lisbon, as passengers will be able to retain their cabins, and meals will be served at regular hours. The *Ophir* will proceed to Tangier and Gibraltar, and will touch at Vigo. The British colony at Oporto are specially anxious to show some civility to the members of the Congress, and arrangements are therefore being made to stay at least one day at Leixoes, either in going or returning.

It is hoped that early application will be made for berths in s.s. *Ophir*, as the accommodation is of necessity limited, and it will be allotted in rotation. The honorary secretaries are Mr. D'Arcy Powei, 10a Chandos Street, Cavendish Square, W., and Dr. Clive Riviere, 19 Devonshire Street, W.

